# Pereira’s Materia Medica

**Published:** 1854-03

**Authors:** 


*Pereira's Materia Medica.*

We have received from the publishers, Blanchard & Lee, of Philadelphia, the second volume of this valuable work. Dr. P. was engaged in revising it with especial reference to the publication of an American edition, at the time of his death. Three-fourths of the entire treatise had passed through his hands. The labor of completing it has devolved on Dr. Alfred S. Taylor and Dr. George Owen Rees, whose names are a sufficient guaranty that the work has been performed with ability. J.

